# Accuracy of the Modified Finnish Diabetes Risk Score (Modified FINDRISC) for detecting metabolic syndrome: Findings from the Indonesian national health survey

**DOI:** 10.1371/journal.pone.0314824

**Published:** 2025-02-12

**Authors:** Indriastuti Cahyaningsih, M. Rifqi Rokhman, Sudikno, Maarten J. Postma, Jurjen van der Schans

**Affiliations:** 1 Department of PharmacoTherapy, Epidemiology and Economics (PTE2), University of Groningen, Groningen, The Netherlands; 2 Faculty of Medicine and Health Sciences, Universitas Muhammadiyah Yogyakarta, Yogyakarta, Indonesia; 3 Faculty of Pharmacy, Universitas Gadjah Mada, Yogyakarta, Indonesia; 4 Research Center for Public Health and Nutrition, National Research and Innovation Agency, Jakarta, Indonesia; 5 Department of Health Sciences, University Medical Center Groningen, University of Groningen, Groningen, The Netherlands; 6 Department of Economics, Econometrics and Finance, Faculty of Economics & Business, University of Groningen, Groningen, The Netherlands; Isawiya General Hospital, Governorate of Gurayyat, SAUDI ARABIA

## Abstract

**Background:**

This study evaluated the diagnostic accuracy of the Modified Finnish Diabetes Risk Score (Modified FINDRISC) for detecting individuals with metabolic syndrome in Indonesia.

**Methods:**

A dataset from the 2018 Indonesian National Basic Health Survey was analysed, and cases of metabolic syndrome were identified in accordance with both National Cholesterol Education Program Adult Treatment Panel III (NCEP-ATP III) and International Diabetes Federation (IDF) guidelines. Diagnostic accuracy of the Modified FINDRISC tool was evaluated using the Area Under the Receiver Operating Characteristic (AUC) curve, while optimal cut-off scores were determined by Youden’s Index.

**Results:**

From 25,432 participants, the mean and standard deviation of the Modified FINDRISC score was 5.7 (SD 4.1). The prevalence of metabolic syndrome was 32.1% and 24.8% based on NCEP-ATP III and IDF criteria, respectively. Based on NCEP-ATP III criteria alone, the AUC of the Modified FINDRISC was 80.9% (80.3%-81.5%) with 74.0% sensitivity and 75.5% specificity. Similarly, based on IDF criteria, AUC was 88.9% (88.5%-89.3%) with 89.8% sensitivity and 75.8% specificity. The optimal cut-off score was 6 for both criteria, with 41.2% of the total participants above the cut-off who would require further confirmation tests.

**Conclusion:**

Metabolic syndrome is prevalent in Indonesia, and the Modified FINDRISC tool offers good diagnostic accuracy for detecting such cases. Utilising Modified FINDRISC as a first-instance screening modality will reduce the number of people requiring further confirmation tests. Modified FINDRISC has the potential for use in daily clinical practice, and the cost-effectiveness of Modified FINDRISC should be further evaluated.

## Introduction

Non-communicable diseases such as cardiovascular disease and diabetes are often underpinned by metabolic syndrome, causing major socioeconomic and healthcare problems worldwide [1]. Metabolic syndrome is indicated by a cluster of cardiometabolic risk factors such as central (abdominal) obesity, elevated blood pressure, high blood triglycerides, high fasting plasma glucose levels, and low levels of high-density lipoprotein (HDL) cholesterol [2, 3]. The global prevalence of metabolic syndrome varies from approximately 12.5% to 31.4% depending on the criteria used for diagnosis. The prevalence of metabolic syndrome in a country increases along with its income level growth [4]. Individuals with metabolic syndrome have a significantly higher risk of mortality compared to those without the condition. They also face a higher risk of developing diabetes and cardiovascular diseases, as well as experiencing cardiovascular and cerebrovascular events such as myocardial infarction and stroke [5–8].

The main risk factor for metabolic syndrome is an unhealthy lifestyle, which is a modifiable risk factor [9]. A healthier lifestyle that includes regular exercise, a healthy diet, a reduction of excess weight, and smoking cessation is recommended for people with metabolic syndrome [10]. Two previous meta-analyses revealed that lifestyle modification programs and pharmacological interventions reduced the risk factors of metabolic syndrome [9, 11]. Therefore, early detection of at-risk individuals is crucial to enable the implementation of risk-lowering measures before cardiovascular diseases and diabetes develop. Generally, the diagnosis of metabolic syndrome is often performed by measuring five clinical and biochemical parameters, namely waist circumference, blood pressure, triglycerides, HDL cholesterol level, and fasting plasma glucose level [2, 3]. However, this method of diagnostic testing is likely not cost-effective and unsuitable in many countries due to practical challenges, especially in low- and middle-income countries [12]. Therefore, a more non-invasive alternative might be appropriate for such settings.

The Finnish Diabetes Risk Score (FINDRISC) tool is a simple, non-invasive, and valid questionnaire that was initially developed for a cohort study with 10-year follow-up for the detection of individuals within the age range of 35–64 years [13]. The International Diabetes Foundation (IDF) has adopted the FINDRISC tool to predict individuals’ risk of developing diabetes [14]. While some studies evaluated the performance of FINDRISC for detecting prediabetes, dysglycaemia, and undiagnosed diabetes [15–17], later studies conducted in Iran [12], Ghana [18], Belgium [19], Lebanon [20], and Greece [21] extended the use of FINDRISC to detect individuals with metabolic syndrome. These studies demonstrate the potential of this non-invasive approach and its simple risk score assessment for early detection of metabolic syndrome. However, studies with different target populations found different diagnostic accuracy of the FINDRISC tool, and each study suggested different optimal cut-off scores. Thus, the generalizability of using FINDRISC to detect metabolic syndrome in other populations is still questionable and requires further assessment.

The actual use of FINDRISC to detect early metabolic syndrome in the Indonesian population requires further evaluation. There has been one earlier study in Indonesia which assessed the performance of Modified FINDRISC in screening individuals with dysglycaemia and undiagnosed diabetes [17]; however, no studies have evaluated the potential of Modified FINDRISC or any other risk assessment tools as an initial screening modality for detecting metabolic syndrome in this country. Therefore, this study aims to examine the diagnostic accuracy of the Modified FINDRISC prediction tool for detecting metabolic syndrome, as well as the prevalence of metabolic syndrome in Indonesia.

## Materials and methods

A cross-sectional analysis was conducted using secondary data collected from the Indonesian Basic Health Research (RISKESDAS) survey conducted in 2018 where the Modified FINDRISC tool was used for detecting individuals with metabolic syndrome. Therefore, ethical approval was not required as no personally identifiable information was utilized. This Indonesian Basic Health Research survey covered all households in 34 provinces and 514 districts.

Permission to access the dataset was obtained from the National Health Development Policy Agency (BKPK), Indonesian Ministry of Health with document number IR.03.01/8/6581/2022 on 20 September 2022. Socio-demographic characteristics and clinical parameters were extracted from this dataset. Socio-demographic characteristics consist of age, gender, educational level, occupation, and residential area. Clinical parameters encompassed body mass index (BMI), waist circumference, systolic and diastolic blood pressures, triglycerides, HDL, low-density lipoprotein (LDL), and total cholesterol, as well as fasting plasma glucose level. All socio-demographic characteristics was based on the participants’ responses, while clinical characteristics were assessed by surveyors in the Indonesian Basic Health Research survey. All data to satisfy responses to the eight Modified FINDRISC risk factor questions was also retrieved from the national health survey dataset.

### Participants

The participants of this study were those participated in the survey who at least measured their fasting plasma glucose level. Individuals younger than 18 years old were excluded.

### Study objectives

The primary objective of this study was to assess the diagnostic accuracy of the Modified FINDRISC for detecting metabolic syndrome, while the secondary objective was to estimate the prevalence of metabolic syndrome in the Indonesian population. Since previous studies indicated that women had a higher prevalence of metabolic syndrome than men [22, 23], we presented the estimates for both women and men separately including the value of 95% of confidence intervals.

### Modified Finnish Diabetes Risk Score (Modified FINDRISC)

The FINDRISC consists of eight risk factors: age, BMI, waist circumference, daily physical activity of at least 30 minutes, dietary consumption of fruits and vegetables, history of taking routine antihypertensive medication, history of hyperglycaemia, and family history of diabetes [13]. Two items of FINDRISC, which are BMI and waist circumference, were directly assessed by surveyors while six other items were based on the participants’ response. All eight items of Modified FINDRISC were similar to the items of original FINDRISC. However, Modified FINDRISC score was calculated using a modified scoring system derived from a previous study in Indonesia in which BMI and waist circumference were classified based on an Asian population [17, 24]. S1 Table describes and compares the differences in scoring between the original FINDRISC and Modified FINDRISC. Modified FINDRISC scores ranged from 0 to 26 points and were classified into low-risk (1–7 points), slightly elevated-risk (7–11 points), moderate-risk (12–14 points), and high-risk of diabetes (more than 14 points).

A previous study which used a longitudinal study design reported the ability of FINDRISC to predict individuals with the risk of developing metabolic syndrome [12]. This study found that individuals with higher FINDRISC score also have a higher risk of developing metabolic syndrome in the future. In addition, the rate ratio of metabolic syndrome is higher for people with higher FINDRISC score even after the age and gender had been adjusted. Therefore, in this study, we assumed that a higher Modified FINDRISC classification should also represent a higher risk for metabolic syndrome.

Six of the eight Modified FINDRISC risk factors namely age, BMI, waist circumference, daily physical activity, history of hyperglycaemia, and family history of diabetes were computed directly from national health survey data. The other two items, ‘dietary consumption of fruits and vegetables’ and ‘history of taking routine antihypertensive medications’, could not be calculated directly due to the way in which the questions were framed. For example, in the national health survey, ‘consumption of fruits’ and ‘consumption of vegetables’ were measured separately by how many days a participant had consumed either food type. Therefore, to obtain the score of dietary consumption of fruits and vegetables in the Modified FINDRISC, we decided to give one point if a participant consumed either one piece of fruit or one vegetable at least once a day. Similarly, to score ‘history of taking routine antihypertensive medication’ in the Modified FINDRISC, we examined three variables: ‘diagnosed with hypertension by a physician’, ‘routine antihypertensive medication’, and ‘blood pressure at time of survey’. Participants received two points if they had been diagnosed with hypertension, had a blood pressure reading over 140/80 mmHg, or were taking routine antihypertensive medication.

### Metabolic syndrome

Metabolic syndrome was evaluated based on two separate guidelines, the NCEP-ATP III and the IDF [2, 3]. To identify participants with metabolic syndrome, these two guidelines suggest that participants be assessed using complete confirmation testing based on five parameters, namely BMI, triglycerides, HDL, blood pressure, and fasting plasma glucose. Based on NCEP-ATP III criteria, participants were considered to have metabolic syndrome if they met at least 3 of the following 5 criteria: (1) central obesity or a waist circumference > 102 cm for men or > 88 cm for women, (2) triglycerides ≥ 150 mg/dl, (3) HDL cholesterol levels < 40 mg/dl for men or < 50 mg/dl for women, (4) blood pressure ≥ 130/85 mmHg or use antihypertensive medications, and (5) fasting plasma glucose ≥ 110 mg/dl or using glucose-lowering medications [3]. In this study, the threshold of fasting plasma glucose was reduced from 110 to 100 mg/dl based on a recommendation by American Diabetes Association (ADA) [25]. The waist circumference threshold was adjusted for an Asian population based on previous studies, with ≥ 90 cm for men and ≥ 80 cm for women [25, 26].

On the other hand, according to IDF criteria, participants were considered to have metabolic syndrome if they experienced central obesity and met at least 2 of the following criteria: (1) triglycerides ≥ 150 mg/dl, (2) HDL < 40 mg/dl for men and < 50 mg/dl for women, (3) systolic blood pressure ≥ 130 mmHg or diastolic blood pressure ≥ 85 mmHg or using antihypertensive medications, and (4) fasting plasma glucose ≥ 100 mg/dl or using glucose-lowering medications [2]. For the purposes of this study, central obesity was defined using ethnic-specific cut-points of waist for Asian populations, and participants were considered to be experiencing central obesity if their waist circumference was at least 90 cm for men and 80 cm for women.

### Data analysis

All variables were visualized by using scatter plots to detect outliers and data entry errors. Participant characteristics were denoted as frequencies (n) and percentages (%) for categorical data, while means and standard deviations (SD) were used for continuous data. Comparison between groups was analysed using Chi-square tests for categorical data, and Mann-Whitney tests for continuous data. Kruskal-Wallis tests were also used to compare different cardiometabolic risk factors based on Modified FINDRISC score classification. The proportion between men and women was analyzed by using one-sample binomial test.

Missing data was predicted by using the Multivariate Imputation by Chained Equations (MICE) package in R software [27, 28]. Ten imputations were set since 8.3% of individuals had missing values. Predictive mean matching (pmm) was used to predict the continuous variables with missing data. After the imputations, these ten imputed data were pooled to calculate the diagnosis accuracy of the Modified FINDRISC and the prevalence of metabolic syndrome. To evaluate the plausibility of the imputations, the prevalence of metabolic syndrome before and after the imputations were compared. The association of cardiometabolic risk factors and Modified FINDRISC classifications was also compared between before and after imputation process.

The accuracy of the Modified FINDRISC prediction tool to detect participants with metabolic syndrome was analysed using the Area Under the Receiver Operating Characteristic (AUC) curve. AUC curves were generated using the pROC package in R software [29]. AUC values between 70% and less than 80% were considered fair, those between 80% and less than 90% as good, and those higher than 90% as excellent [30]. Sensitivity, specificity, positive predictive values, and negative predictive values were calculated based on different cut-offs of the Modified FINDRISC score. The optimal cut-off score was determined using Youden’s Index. All statistical analyses were performed using R software, with the level of statistical significance set at a *p*-value < 0.05.

The potential of Modified FINDRISC as the initial screening modality prior to instituting complete confirmation testing was evaluated using numbers needed to screen (NNS). NNSs were calculated based on the number of participants screened before at least one individual with metabolic syndrome was detected. NNSs were compared by two scenarios. Scenario one was the administration of complete confirmation tests to all participants in the first instance. In scenario two, the Modified FINDRISC prediction tool was applied as an initial screening tool followed by complete confirmation testing for participants obtaining Modified FINDRISC scores higher than the cut-off. Lower NNS values were preferred as they indicate a greater level of efficiency for any screening modality.

## Results

**Fig 1:**
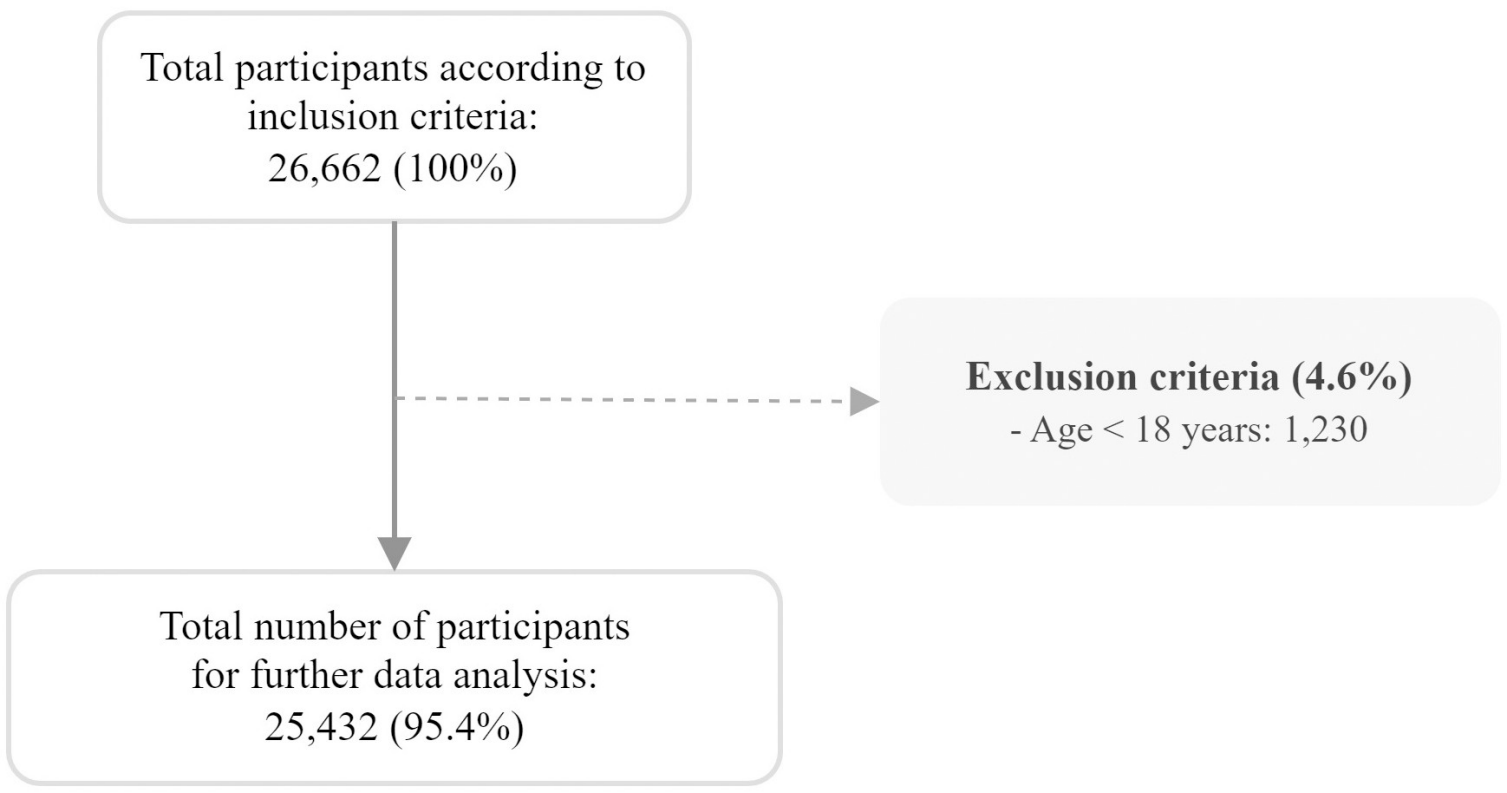
Selection of participants.

The participant selection criteria are presented in Fig 1. From 26,662 participants who met the inclusion criteria, 4.6% of participants were excluded from data analysis because their age was lower than 18 years. Finally, 25,432 (95.4%) participants had complete data for further analysis (S1 Dataset).

Table 1 displays the characteristics of all participants in this study. Among all participants, the mean average age was 45.1 (SD = 14.8) years, elementary school was the highest educational level (54.3%), the mean average BMI was 24.3 (SD = 4.8) kg/m^2^, waist circumference was 80.8 (SD = 12.4) cm, approximately half resided in a rural area (51.6%), and the majority of the participants were women (62.7%). Compared to men, women had significantly higher diastolic blood pressure, HDL, LDL, total cholesterols and fasting plasma glucose level. On the other hand, men had significantly higher triglycerides compared to women. Although on average, the male participants in this study were older, women still had a higher risk for metabolic syndrome across the majority of Modified FINDRISC risk factors compared to men, with BMI, waist circumference, use of antihypertensive medications, and history of hyperglycaemia all being significantly higher. The mean average Modified FINDRISC score of women at 6.2 (SD = 4.2) was significantly higher (*p*<0.001) compared to men at 4.8 (SD = 3.9), while the overall Modified FINDRISC score was 5.7 (SD = 4.1).

**Table 1:** Participants’ characteristics (n = 25,432).

	Overall	Gender		*p*-value
		Men	Women	
Total, n (%)	25,432 (100.0)	9,482 (37.3)	15,950 (62.7)	<0.001
Education, n (%)				
Elementary school	13,813 (54.3)	4,852 (51.2)	8,961 (56.2)	<0.001
High school	10,008 (39.4)	3,941 (41.5)	6,067 (38.0)	
Diploma or higher	1,611 (6.3)	689 (7.3)	922 (5.8)	
Occupation, n (%)				
Unemployed	9,112 (35.8)	998 (10.5)	8,114 (50.9)	<0.001
Student	546 (2.2)	215 (2.3)	331 (2.1)	
Employed	15,774 (62.0)	8,269 (87.2)	7,505 (47.0)	
Rural area, n (%)	13,115 (51.6)	5,080 (53.6)	8,035 (50.4)	<0.001
***Clinical parameters***				
Blood pressure (mg/dL), mean (SD)				
Systolic	132.4 (25.0)	132.2 (22.8)	132.5 (26.3)	0.360
Diastolic	84.2 (13.3)	82.8 (12.9)	85.0 (13.5)	<0.001
Individuals with hypertension, n (%)	6,180 (24.5)	1,948 (20.8)	4,232 (26.7)	<0.001
Triglycerides (mg/dL), mean (SD)	122.9 (86.2)	134.5 (98.1)	115.9 (77.5)	<0.001
High-density lipoprotein (mg/dL), mean (SD)	48.9 (11.4)	45.1 (10.2)	51.2 (11.4)	<0.001
Low-density lipoprotein (mg/dL), mean (SD)	125.1 (34.4)	121.5 (32.9)	127.3 (35.1)	<0.001
Total cholesterol (mg/dL), mean (SD)	185.3 (40.2)	178.9 (39.0)	189.2 (40.5)	<0.001
Fasting plasma glucose (mg/dL), mean (SD)	102.9 (33.2)	101.4 (27.8)	103.8 (36.0)	<0.001
***Modified FINDRISC components***				
Age (years), mean (SD)	45.1 (14.8)	46.8 (15.2)	44.2 (14.4)	<0.001
Body mass index (kg/m^2^), mean (SD)	24.3 (4.8)	22.8 (4.2)	25.1 (5.0)	<0.001
Waist circumference (cm), mean (SD)	80.8 (12.4)	79.7 (12.2)	81.4 (12.4)	<0.001
Daily physical activity, no, n (%)	5,443 (21.4)	3,204 (33.8)	2,239 (14.0)	<0.001
Fruits and vegetables consumption, no, n (%)	9,510 (37.4)	3,923 (41.4)	5,587 (35.0)	<0.001
Antihypertensive medication, yes, n (%)	6,180 (24.5)	1,948 (20.8)	4,232 (26.7)	<0.001
History of hyperglycaemia, yes, n (%)	1,172 (4.6)	421 (4.4)	751 (4.7)	0.339
Family with diabetes, yes, n (%)	1,006 (4.0)	433 (4.6)	573 (3.6)	<0.001
Modified FINDRISC score, mean (SD)	5.7 (4.1)	4.8 (3.9)	6.2 (4.2)	<0.001

*Notes*. FINDRISC, Finnish diabetes risk score; SD, standard deviation.

In general, individuals with higher Modified FINDRISC scores were older and had higher BMI, waist circumference, blood pressure, triglycerides, LDL cholesterol, total cholesterol, and fasting plasma glucose levels, and lower HDL cholesterol (Table 2). These findings indicate that individuals in higher Modified FINDRISC classifications are at higher risk of metabolic syndrome. The prevalence of metabolic syndrome was higher as the Modified FINDRISC risk classification increased, irrespective of whether it was based on NCEP-ATP III or IDF criteria. The association of cardiometabolic risk factors and Modified FINDRISC classifications after the imputation was similar to the association before the imputation (S2 Table).

**Table 2:** Association of cardiometabolic risk factors and Modified FINDRISC classifications.

	Modified FINDRISC classification, mean (SD)				*p*-value
	Low-risk	Slightly elevated-risk	Moderate-risk	High-risk	
Total, n (%)	14,947 (58.8)	8,150 (32.0)	1,676 (6.6)	659 (2.6)	<0.001
Age (years)	41.9 (14.7)	48.0 (13.8)	55.1 (11.3)	58.4 (10.0)	<0.001
Body mass index (kg/m^2^)	21.8 (3.0)	27.4 (4.9)	28.7 (4.5)	29.0 (4.0)	<0.001
Waist circumference (cm)	74.3 (8.3)	88.8 (11.2)	93.7 (10.9)	95.8 (9.9)	<0.001
Blood pressure (mg/dL)					
Systolic	125.4 (21.0)	138.9 (25.6)	154.3 (28.9)	154.1 (26.1)	<0.001
Diastolic	80.5 (11.5)	88.2 (13.5)	93.8 (15.2)	91.8 (14.3)	<0.001
Triglycerides (mg/dL)	109.2 (74.0)	136.5 (92.0)	159.5 (111.2)	170.6 (107.0)	<0.001
High-density lipoprotein (mg/dL)	49.7 (11.5)	47.9 (11.1)	47.5 (11.0)	46.9 (11.0)	<0.001
Low-density lipoprotein (mg/dL)	119.0 (32.5)	131.7 (34.7)	141.1 (36.3)	144.3 (39.2)	<0.001
Total cholesterol (mg/dL)	177.6 (37.9)	193.4 (40.0)	206.3 (42.8)	210.6 (47.3)	<0.001
Fasting plasma glucose (mg/dL)	97.5 (22.0)	105.9 (36.6)	121.5 (54.2)	142.6 (65.4)	<0.001
Metabolic syndrome, n (%)					
NCEP-ATP III	2,233 (14.9)	4,559 (55.9)	1,239 (73.9)	561 (85.1)	<0.001
IDF	674 (4.5)	4,188 (51.4)	1,194 (71.2)	552 (83.8)	<0.001

*Notes*. NCEP-ATP III, National Cholesterol Education Program Adult Treatment Panel III; IDF, International Diabetes Federation; SD, standard deviation.

In the dataset, there were eight variables had missing data (S3 Table). Four variables had missing data less than 5%, which were BMI (0.5%), waist circumference (1.3%), systolic, and diastolic blood pressure (2.1%), while four other variables had the same 6.3% of missing data, namely triglycerides, HDL, LDL, and total cholesterol.

### Metabolic syndrome

The percentage of individuals with metabolic syndrome was approximately 32.1% (31.5%-32.7%) based on NCEP-ATP III criteria, and 24.8% (24.3%-25.4%) based on IDF criteria (Table 3). Both sets of guidelines found that women had a significantly higher prevalence of metabolic syndrome compared to men, although IDF criteria reported a 19.2% difference between the genders compared to the NCEP-ATP III criteria at only 13.7%. In addition, the prevalence of metabolic syndrome before and after imputation showed only slightly different with a difference lower than 2% (S4 Table).

**Table 3:** Prevalence of metabolic syndrome in Indonesia.

	Percentage (95% CI)			*p*-value
	Overall	Men	Women	
NCEP-ATP III	32.1 (31.5–32.7)	23.5 (22.7–24.4)	37.2 (36.4–37.9)	<0.001
IDF	24.8 (24.3–25.4)	12.8 (12.1–13.5)	32.0 (31.3–32.7)	<0.001

*Notes*. NCEP-ATP III, National Cholesterol Education Program Adult Treatment Panel III; IDF, International Diabetes Federation; 95% CI, 95% of confidence intervals.

**Fig 2:**
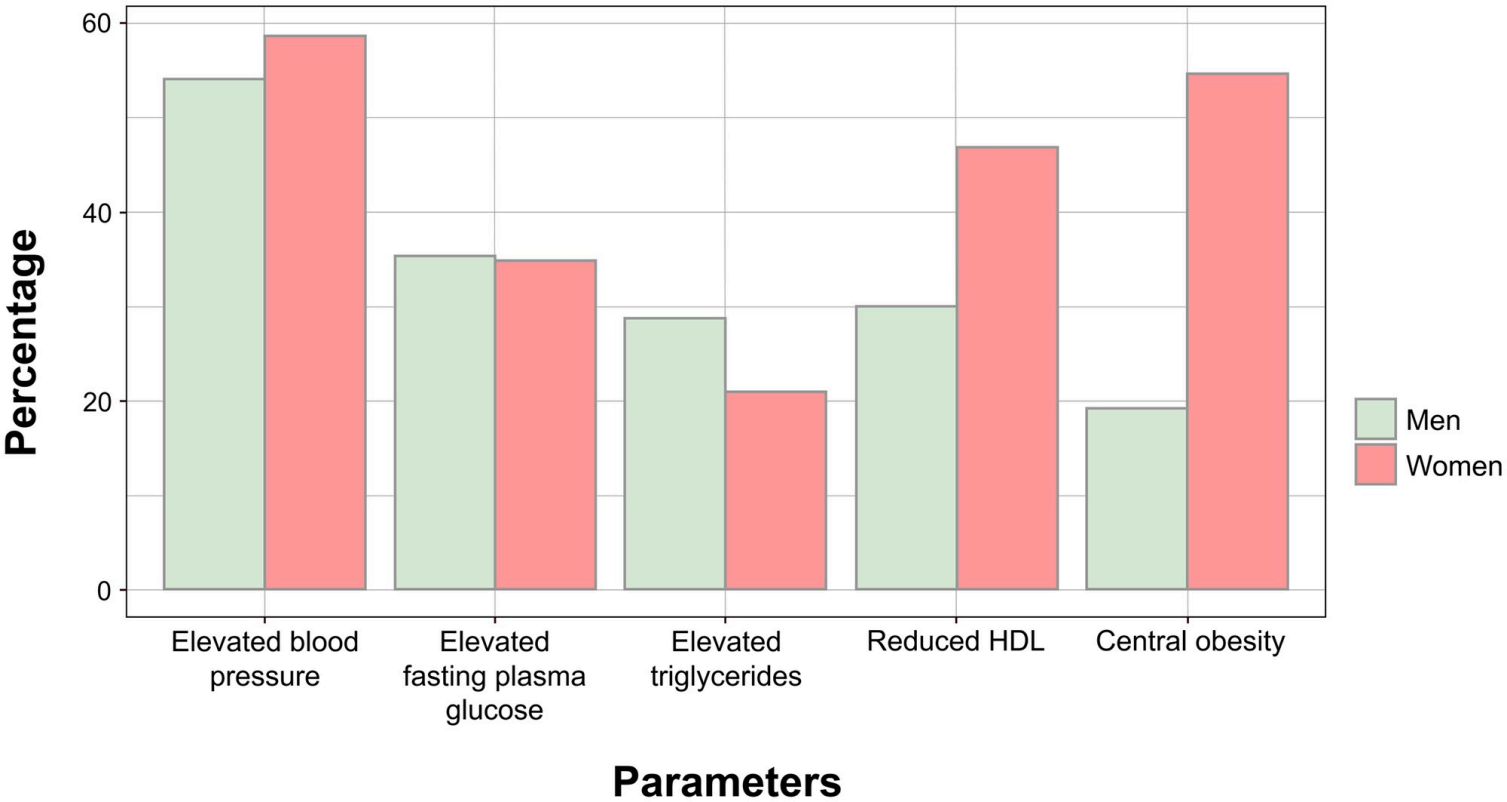
The percentage of participants experiencing metabolic syndrome risk factors based on gender. *Notes*. HDL, high-density lipoprotein.

In total, 8,592 (33.8%) participants were identified as having metabolic syndrome based on either the NCEP-ATP III or IDF criteria. From this number, 6,608 (76.9%) participants were detected by both criteria, while 1,984 (23.1%) were detected only by NCEP-ATP III criteria. All participants with metabolic syndrome detected using IDF criteria were also detected using NCEP-ATP III. The characteristics of participants detected by NCEP-ATP III criteria only, IDF criteria only, and both criteria are presented in S5 Table. Fig 2 compares the percentage of individuals experiencing metabolic syndrome risk factors based on gender. The percentage of women experiencing metabolic risk factors was higher than that of men across three parameters: center obesity, reduced HDL, and elevated blood pressure. Only in elevated triglycerides, men had a higher percentage than women, and in elevated fasting plasma glucose, men also had slightly higher percentage than women.

### Diagnostic accuracy

**Fig 3:**
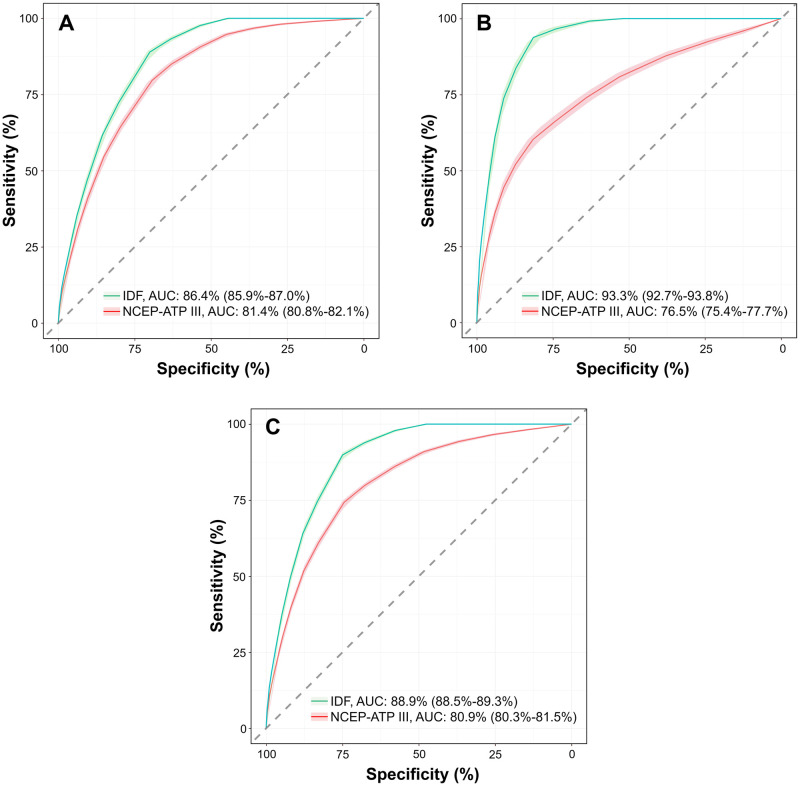
Receiver operating characteristic curves of Modified FINDRISC for detecting metabolic syndrome in women (A), men (B), and total participants (C). *Notes*. AUC, area under the receiver operating characteristic curve; IDF, International Diabetes Federation; NCEP-ATP III, National Cholesterol Education Program Adult Treatment Panel III (NCEP-ATP III).

Based on IDF criteria, the AUC of Modified FINDRISC for detecting metabolic syndrome in total participants was 88.9% (88.5%-89.3%), while the AUC was 80.9% (80.3%-81.5%) based on NCEP-ATP III criteria. Both values are considered good in terms of diagnostic accuracy (Fig 3). The AUC of the Modified FINDRISC for detecting metabolic syndrome based on IDF criteria was higher compared to the NCEP-ATP III criteria in all participants, both men and women.

Table 4 presents the performance of Modified FINDRISC based on different cut-offs. Based on the NCEP-ATP III, the optimal cut-off was 6 with 74.0% sensitivity and 75.5% specificity. Applying Modified FINDRISC as the initial screening modality resulted in detection of 6,359 individuals (74.0%) with metabolic syndrome, with only 41.2% of the total population above the cut-off who required further confirmation tests. Compared to complete confirmation testing, the NNS value decreased from 3.1 to 1.6, resulting in a lower number of screenings to detect one individual with metabolic syndrome. Similarly, based on the IDF criteria, the optimal cut-off was 6 with 89.8% sensitivity and 75.8% specificity. Applying Modified FINDRISC as the initial screening modality resulted in detection of 5,934 individuals (89.8%) with metabolic syndrome, and a decrease in NNS value from 4.0 to 1.8 compared to applying complete confirmation testing to all participants. Complete data regarding different Modified FINDRISC cut-off scores can be found in S6 Table.

**Table 4:** Different cut-offs and Modified FINDRISC diagnostic accuracy for detecting individuals with metabolic syndrome.

	Cut-off	Sensitivity (%)	Specificity (%)	PPV (%)	NPV (%)	Youden’s index	Case above the cut-off (%)	Case detected n (%)	NNS	NNS by screening all participants
NCEP-ATP III	5	79.6	68.7	56.5	86.9	0.48	47.6	6,841 (79.6)	1.8	3.1
	**6**	**74.0**	**75.5**	**60.6**	**85.1**	**0.50**	**41.2**	**6,359 (74.0)**	**1.6**	
	7	60.4	83.7	65.3	80.5	0.44	31.2	5,190 (60.4)	1.5	
IDF	5	93.9	68.6	51.2	97.0	0.63	47.6	6,202 (93.9)	2.0	4.0
	**6**	**89.8**	**75.8**	**56.6**	**95.5**	**0.66**	**41.2**	**5,934 (89.8)**	**1.8**	
	7	74.2	83.8	61.7	90.2	0.58	31.2	4,900 (74.2)	1.6	

*Notes*. NCEP-ATP III, National Cholesterol Education Program Adult Treatment Panel III; IDF, International Diabetes Federation; PPV, positive predictive value; NPV, negative predictive value; NNS, number needed to screen.

In addition, the largest proportion of participants was in 35–64 age group with 66.1% of total participants, while only 23.7% and 10.2% were in <35 age group and >64 age group, respectively. The comparison of diagnostic accuracy of Modified FINDRISC to detect metabolic syndrome in different age groups can be found in S7 Table. Across three different age groups, the diagnostic accuracy ranged from 77.6% in age >64 age group to 82.1% in <35 age group based on NCEP-ATP III, while based on IDF, the diagnostic accuracy ranged from 88.2% in 35–64 age group to 92.7% in >64 age group.

## Discussion

This is the first study to use a large national dataset to assess the application of the Modified FINDRISC prediction tool for detecting metabolic syndrome in Indonesia. Our study findings indicate that the Modified FINDRISC can predict individuals at high risk for metabolic syndrome with good diagnostic accuracy (AUC values over 80%). The use of Modified FINDRISC as an initial screening test can reduce the number of individuals requiring further confirmation testing to detect individuals with metabolic syndrome. The Modified FINDRISC approach provides a more efficient screening process.

In this study, the accuracy of the Modified FINDRISC tool for detecting metabolic syndrome is higher than in previous studies. For comparison, the AUC of FINDRISC for detecting metabolic syndrome was 65% in the study from Iran [12], 69% in Ghana [18], 69% in Belgium [19], 71% in Lebanon [20], and 73% in Greece [21]. However, these findings may simply highlight the fact that FINDRISC diagnostic accuracy may differ across populations of different ethnic backgrounds [12]. Therefore, prior to application, FINDRISC or its modified version should first be evaluated for diagnostic accuracy in the target population.

The optimal cut-off score in this study is lower than in other studies evaluating the use of FINDRISC for metabolic syndrome screening. Other studies reported cut-off scores of 10.5 points in Lebanon [20], 12 points in Iran [12], and 15 points in Greece [21]. This is not unusual, however, as the mean of the Modified FINDRISC score in our sample at 5.5 (SD 4.1) points was also lower compared to the mean of the FINDRISC scores identified in Lebanon at 10 (SD 4.2) points [20], Iran 11.2 (SD 3.8) points [12], and Greece 12.6 (SD 4.9) points [21]. Although the cut-off score in this study was lower, the percentage of cases above the cut-off score in this study is quite close to another study undertaken in Greece [21]. In addition, the sensitivity and specificity in this study were also higher than those in other studies [12, 20, 21]. If similar higher cut-off scores were applied in Indonesia, fewer individuals would require further confirmation testing due to increased specificity. However, a higher cut-off score will also decrease the sensitivity of the Modified FINDRISC instrument to a point where a fewer number of true positive cases of metabolic syndrome will be detected.

Based on the recommended cut-off of 6 points in our study, the majority of participants in the low-risk group (1–7 points) were not recommended for further screening, as only a few people in this group had a Modified FINDRISC score of 7 points. On the contrary, all participants in the slightly elevated-risk group should undergo further examination since they had higher Modified FINDRISC score than the cut-off. This approach is beneficial to minimize the confirmation test since the low-risk group had large number of participants (58.7% of total) but low prevalence of metabolic syndrome, while other groups had lower number of participants but higher prevalence.

Along with an increase in Modified FINDRISC classification, cardiometabolic and diabetes risk factors such as BMI, waist circumference, systolic and diastolic blood pressure, triglycerides, HDLs, LDLs, total cholesterol, and fasting plasma glucose also increase. This finding aligns with previous studies [31]. In a cohort study design, FINDRISC score higher than 12 points is associated with a 4.1 times increased risk of diabetes and 1.6 times increased risk of cardiovascular disease [32]. In our study, the prevalence of metabolic syndrome also increases with the increase of Modified FINDRISC classification. This indicates that the non-invasive approach of using a risk score, such as that of the Modified FINDRISC tool, may not only be potentially beneficial in the detection of metabolic syndrome, but also in detecting individuals at high risk of developing cardiovascular disease and diabetes.

Based on IDF criteria, the overall prevalence of metabolic syndrome is slightly lower than the prevalence based on NCEP-ATP III criteria. A previous study comparing 5 criteria of metabolic syndrome indicated that the performance of IDF screening criteria had the highest level of agreement with results based on WHO criteria [33]. Therefore, compared to NCEP ATP-III, the revised National Cholesterol Education Program (NCEP-R) or the American Association of Clinical Endocrinologists (AACE) criteria, this previous study concluded that IDF criteria are the recommended criteria for application [33]. In our study, the Modified FINDRISC tool demonstrated good diagnostic accuracy in detecting individuals with metabolic syndrome using either the NCEP-ATP III or IDF criteria.

### Study implications

This study has found a substantial prevalence of metabolic syndrome in the Indonesian population. Our finding is higher to those in previous studies, which reported the prevalence of metabolic syndrome at approximately 20% in the Asia-Pacific region [34]; 25% in the Middle-East region [35], and 25% globally [36]. A previous study evaluating metabolic syndrome in Indonesia found that the prevalence had reached 21.7% in 2018 [37], although this study did not take into account elevated triglycerides. The high prevalence in our study highlights the urgency for intervention programs because metabolic syndrome is a precursor toward increased risk for developing cardiovascular diseases and diabetes. During the National Health Survey, participants with positive screening results were advised to visit the nearest primary healthcare facility for further assessment and treatment by a physician. However, there was no monitoring of how many participants visited primary healthcare or underwent further assessments as follow-up for their health conditions. We recommend linking the National Health Survey screening results to National Health Insurance to evaluate participants’ disease progression.

A previous study has shown that a one-year lifestyle intervention program consisting of six bi-monthly sessions with a dietician for individuals at high risk of developing T2D was significantly associated with a lower FINDRISC score, a lower triglyceride level, and a higher baseline HDL-level [38]. Another study indicated that, although online health services and related digital technologies improved specific anthropometric outcomes, they did not affect biochemical indicators of metabolic syndrome [39]. Further studies are needed to evaluate the effectiveness of programs aimed at reducing the risk of metabolic syndrome in Indonesia. Additionally, since our study found a higher prevalence of metabolic syndrome in women—likely due to the higher percentage of women with central obesity—tailoring direct interventions for women is important and should be prioritized.

Another positive implication of adopting the Modified FINDRISC as an initial screening tool for detecting metabolic syndrome is its efficiency compared to directly measuring all clinical parameters according to NCEP-ATP III or IDF criteria. Based on both criteria, our study revealed that only 41.2% of people above the cut-off score required further confirmation testing, while 74.0% and 89.8% of individuals determined to have metabolic syndrome according to NCEP-ATP III and IDF criteria respectively were able to be detected. Besides detecting individuals with metabolic syndrome, the Modified FINDRISC screening approach will also detect individuals with undiagnosed type 2 diabetes, as previous studies have documented that individuals with high Modified FINDRISC scores will have a higher-risk of undiagnosed type 2 diabetes [15–17, 40]. Therefore, we suggest that future studies evaluate the cost-effectiveness of using the Modified FINDRISC as an initial screening tool for metabolic syndrome followed by further examination. Moreover, a cost-effectiveness study is needed to determine which age group should be prioritized for screening, as this depends on the prevalence of the target group and the cost of screening [41].

This study has several limitations. First, although multistage random sampling was used, the number of participants with a family history of diabetes was too low, considering that a previous study found that one-third of participants had relatives with diabetes [17]. In addition, during the national Indonesian Basic Health Research survey, participants were only asked if they had family members with diabetes, without specifying whether these were first- or second-degree relatives. As a result, we allocated 5 points for family members with diabetes regardless of whether the relative was of the first or second degree. Secondly, data was evaluated as an aggregate data nationally and was not analyzed based on regions or provinces. We encourage future studies to address these issues and evaluate the distribution of prevalence in Indonesia. Lastly, six out of eight items in the Modified FINDRISC are self-reported, which may lead to recall bias. However, a previous study has confirmed the diagnostic accuracy of the Modified FINDRISC for screening individuals with prediabetes and undiagnosed diabetes in Indonesia [17].

## Conclusions

Metabolic syndrome has a substantial prevalence in Indonesia, and the Modified FINDRISC prediction tool has demonstrated good diagnostic accuracy in detecting individuals with metabolic syndrome. Applying Modified FINDRISC as an initial screening measure will reduce the number of people requiring further confirmation tests, making screening and diagnosis more efficient. Therefore, Modified FINDRISC has the potential for use in daily clinical practice, and the cost-effectiveness of this approach should be further evaluated.

## Supporting information

S1 Dataset (XLSX)

S1 Table Scoring of FINDRISC-BI and Modified FINDRISC-BI. (DOCX)

S2 Table Comparison of association between cardiometabolic risk factors and Modified FINDRISC classifications before and after imputation. (DOCX)

S3 Table Number of participants with missing data. (DOCX)

S4 Table Comparison of prevalence of metabolic syndrome before and after imputation. (DOCX)

S5 Table Characteristics of participants detected by NCEP-ATP III criteria only, IDF criteria only, and both criteria. (DOCX)

S6 Table Different cut-offs and diagnostic accuracy of Modified FINDRISC to detect individuals with metabolic syndrome in Indonesia. (DOCX)

S7 Table Comparison of diagnostic accuracy of Modified FINDRISC to detect metabolic syndrome in different age groups. (DOCX)

S1 File Research protocol. (DOCX)

S1 Checklist STROBE statement. (DOCX)

S2 Checklist Inclusivity in global research. (DOCX)
