# Supplementary material for: Accuracy of the Modified Finnish Diabetes Risk Score (Modified FINDRISC) for detecting metabolic syndrome: Findings from the Indonesian national health survey
Source: PLoS One. 2025 Feb 12;20(2):e0314824. doi: 10.1371/journal.pone.0314824 (PMC11819590; doi:10.1371/journal.pone.0314824)
Supplement: S1 Table — (DOCX) [file pone.0314824.s002.docx]

**S1 Table.** Scoring of FINDRISC and Modified FINDRISC

|  | FINDRISC* | Modified FINDRISC* |
| --- | --- | --- |
| Age |  |  |
| <45 years | 0 points | 0 points |
| 45-54 years | 2 points | 2 points |
| 55-64 years | 3 points | 3 points |
| >64 years | 4 points | 4 points |
| Body mass index |  |  |
| <25 kg/m^2^ | 0 points | 0 points (<25 kg/m^2^) |
| 25-30 kg/m^2^ | 1 point | 1 point (25-27 kg/m^2^) |
| >30 kg/m^2^ | 3 points | 3 points (>27 kg/m^2^) |
| Waist circumference |  |  |
| Female <80 cm, male <94 cm | 0 points | 0 points (Female <80 cm, male <90 cm) |
| Female 80-88 cm, male 94-102 cm | 3 points | - |
| Female >88 cm, male >102 cm | 4 points | 4 points (Female >80 cm, male >90 cm) |
| Physical activity (30 minutes/day) |  |  |
| Yes | 0 points | 0 points |
| No | 2 points | 2 points |
| Fruit and vegetable consumption |  |  |
| Every day | 0 points | 0 points |
| Not every day | 1 point | 1 point |
| Antihypertensive medication |  |  |
| No | 0 points | 0 points |
| Yes | 2 points | 2 points |
| History of high blood glucose |  |  |
| No | 0 points | 0 points |
| Yes | 5 points | 5 points |
| Family members with diabetes |  |  |
| No | 0 points | 0 points |
| Grandparent, aunt, uncle or first cousin | 3 points | 3 points |
| Parent, brother, sister or own child | 5 points | 5 points |

*Notes:*

The difference between the FINDRISC and Modified FINDRISC is based on the scores for the body mass index and waist circumference categories.

*Adapted with permission from Jaana Lindström, MSC and Jaakko Tuomilehto, MD, PHD. Copyright 2003 by the American Diabetes Association.

FINDRISC was translated into Bahasa Indonesia by Rokhman MR, Arifin B, Zulkarnain Z, et al. Translation and performance of the Finnish Diabetes Risk Score for detecting undiagnosed diabetes and dysglycaemia in the Indonesian population. *PLoS One* 2022; 17: 1–16. doi: 10.1371/journal.pone.0269853
