# Supplementary material for: Accuracy of the Modified Finnish Diabetes Risk Score (Modified FINDRISC) for detecting metabolic syndrome: Findings from the Indonesian national health survey
Source: PLoS One. 2025 Feb 12;20(2):e0314824. doi: 10.1371/journal.pone.0314824 (PMC11819590; doi:10.1371/journal.pone.0314824)
Supplement: S2 Table — (DOCX) [file pone.0314824.s003.docx]

**S2 Table.** Comparison of association between cardiometabolic risk factors and Modified FINDRISC classifications before and after imputation

|  | | Modified FINDRISC classification, mean (SD) | | | | | *p*-value | |
| --- | --- | --- | --- | --- | --- | --- | --- | --- |
|  |  | Low-risk | Slightly elevated-risk | Moderate-risk | High-risk | |  |  |
| ***Before imputation*** | | | | | | | |  |
| n (%) | | 14,614 (58.7) | 7,978 (32.1) | 1,636 (6.6) | 644 (2.6) | <0.001 | |  |
|  | |  |  |  |  |  | |  |
| Age (years) | | 41.9 (14.6) | 47.9 (13.7) | 55.1 (11.1) | 58.3 (10.0) | <0.001 | |  |
| Body mass index (kg/m^2^) | | 21.8 (3.0) | 27.4 (4.9) | 28.7 (4.5) | 29.1 (4.0) | <0.001 | |  |
| Waist circumference (cm) | | 74.3 (8.2) | 88.8 (11.1) | 93.7 (10.9) | 95.9 (9.9) | <0.001 | |  |
| Blood pressure (mg/dL) | |  |  |  |  |  | |  |
| Systolic | | 125.4 (20.9) | 138.8 (25.6) | 154.1 (28.6) | 154.4 (26.1) | <0.001 | |  |
| Diastolic | | 80.5 (11.5) | 88.2 (13.4) | 93.7 (15.2) | 91.8 (14.4) | <0.001 | |  |
| Triglycerides (mg/dL) | | 108.7 (74.4) | 136.0 (91.7) | 160.8 (111.5) | 172.0 (108.7) | <0.001 | |  |
| High-density lipoprotein (mg/dL) | | 49.6 (11.5) | 48.0 (11.1) | 47.6 (10.9) | 47.0 (11.1) | <0.001 | |  |
| Low-density lipoprotein (mg/dL) | | 118.7 (32.1) | 131.7 (34.3) | 142.0 (36.2) | 144.6 (39.4) | <0.001 | |  |
| Total cholesterol (mg/dL) | | 177.3 (37.4) | 193.3 (39.4) | 207.3 (42.8) | 210.9 (47.5) | <0.001 | |  |
| Fasting plasma glucose (mg/dL) | | 97.4 (21.9) | 106.0 (36.8) | 121.6 (54.4) | 142.4 (64.2) | <0.001 | |  |
|  | |  |  |  |  |  | |  |
| Metabolic syndrome, n (%) | |  |  |  |  |  | |  |
| NCEP-ATP III | | 2,059 (14.1) | 4,269 (53.5) | 1,177 (71.9) | 545 (84.6) | <0.001 | |  |
| IDF | | 632 (4.3) | 3,941 (49.4) | 1,141 (69.7) | 537 (83.4) | <0.001 | |  |
|  | |  |  |  |  |  | |  |
| ***After imputation*** | | | | | | | |  |
| n (%) | 14,947 (58.8) | | 8,150 (32.0) | 1,676 (6.6) | 659 (2.6) | | <0.001 | |
|  |  | |  |  |  | |  | |
| Age (years) | 41.9 (14.7) | | 48.0 (13.8) | 55.1 (11.3) | 58.4 (10.0) | | <0.001 | |
| Body mass index (kg/m^2^) | 21.8 (3.0) | | 27.4 (4.9) | 28.7 (4.5) | 29.0 (4.0) | | <0.001 | |
| Waist circumference (cm) | 74.3 (8.3) | | 88.8 (11.2) | 93.7 (10.9) | 95.8 (9.9) | | <0.001 | |
| Blood pressure (mg/dL) |  | |  |  |  | |  | |
| Systolic | 125.4 (21.0) | | 138.9 (25.6) | 154.3 (28.9) | 154.1 (26.1) | | <0.001 | |
| Diastolic | 80.5 (11.5) | | 88.2 (13.5) | 93.8 (15.2) | 91.8 (14.3) | | <0.001 | |
| Triglycerides (mg/dL) | 109.2 (74.0) | | 136.5 (92.0) | 159.5 (111.2) | 170.6 (107.0) | | <0.001 | |
| High-density lipoprotein (mg/dL) | 49.7 (11.5) | | 47.9 (11.1) | 47.5 (11.0) | 46.9 (11.0) | | <0.001 | |
| Low-density lipoprotein (mg/dL) | 119.0 (32.5) | | 131.7 (34.7) | 141.1 (36.3) | 144.3 (39.2) | | <0.001 | |
| Total cholesterol (mg/dL) | 177.6 (37.9) | | 193.4 (40.0) | 206.3 (42.8) | 210.6 (47.3) | | <0.001 | |
| Fasting plasma glucose (mg/dL) | 97.5 (22.0) | | 105.9 (36.6) | 121.5 (54.2) | 142.6 (65.4) | | <0.001 | |
|  |  | |  |  |  | |  | |
| Metabolic syndrome, n (%) |  | |  |  |  | |  | |
| NCEP-ATP III | 2,233 (14.9) | | 4,559 (55.9) | 1,239 (73.9) | 561 (85.1) | | <0.001 | |
| IDF | 674 (4.5) | | 4,188 (51.4) | 1,194 (71.2) | 552 (83.8) | | <0.001 | |

*Notes.* NCEP-ATP III, National Cholesterol Education Program Adult Treatment Panel III; IDF, International Diabetes Federation; SD, standard deviation.
