# Supplementary material for: Accuracy of the Modified Finnish Diabetes Risk Score (Modified FINDRISC) for detecting metabolic syndrome: Findings from the Indonesian national health survey
Source: PLoS One. 2025 Feb 12;20(2):e0314824. doi: 10.1371/journal.pone.0314824 (PMC11819590; doi:10.1371/journal.pone.0314824)
Supplement: S3 Table — (DOCX) [file pone.0314824.s004.docx]

**S3 Table.** Number of participants with missing data

| Variable | n (%) |
| --- | --- |
| Body mass index | 130 (0.5) |
| Waist circumference | 318 (1.3) |
| Blood pressure |  |
| Systolic | 542 (2.1) |
| Diastolic | 542 (2.1) |
| Triglycerides | 1,598 (6.3) |
| High-density lipoprotein | 1,598 (6.3) |
| Low-density lipoprotein | 1,598 (6.3) |
| Total cholesterol | 1,598 (6.3) |
