# Supplementary material for: Accuracy of the Modified Finnish Diabetes Risk Score (Modified FINDRISC) for detecting metabolic syndrome: Findings from the Indonesian national health survey
Source: PLoS One. 2025 Feb 12;20(2):e0314824. doi: 10.1371/journal.pone.0314824 (PMC11819590; doi:10.1371/journal.pone.0314824)
Supplement: S4 Table — (DOCX) [file pone.0314824.s005.docx]

**S4 Table.** Comparison of prevalence of metabolic syndrome between before and after imputation

|  | Prevalence (95% CI) | | | *p*-value |
| --- | --- | --- | --- | --- |
|  | Overall | Men | Women |  |
| ***Before imputation*** |  |  |  |  |
| NCEP-ATP III | 32.1 (31.5-32.7) | 23.5 (22.7-24.4) | 37.2 (36.4-37.9) | <0.001 |
| IDF | 24.8 (24.3-25.4) | 12.8 (12.1-13.5) | 32.0 (31.3-32.7) | <0.001 |
|  |  |  |  |  |
| ***After imputation*** |  |  |  |  |
| NCEP-ATP III | 33.7 (33.2-34.3) | 24.6 (23.8-25.5) | 39.1 (38.4-39.9) | <0.001 |
| IDF | 26.0 (25.5-26.5) | 13.3 (12.7-14.0) | 33.5 (32.8-34.2) | <0.001 |

*Notes.* NCEP-ATP III, National Cholesterol Education Program Adult Treatment Panel III; IDF, International Diabetes Federation; SD, standard deviation.
