# Supplementary material for: Accuracy of the Modified Finnish Diabetes Risk Score (Modified FINDRISC) for detecting metabolic syndrome: Findings from the Indonesian national health survey
Source: PLoS One. 2025 Feb 12;20(2):e0314824. doi: 10.1371/journal.pone.0314824 (PMC11819590; doi:10.1371/journal.pone.0314824)
Supplement: S5 Table — (DOCX) [file pone.0314824.s006.docx]

**S5 Table.** Characteristics of participants detected by NCEP-ATP III criteria only, IDF criteria only, and both criteria

|  | Participants detected by | | |
| --- | --- | --- | --- |
|  | NCEP-ATP III | IDF | Both criteria |
| Total, n (%) | 8,592 (33.8) | 6,608 (26.0) | 6,608 (26.0) |
| Education, n (%) |  |  |  |
| Elementary school | 4,967 (57.8) | 3,653 (55.3) | 3,653 (55.3) |
| High school | 3,071 (35.7) | 2,494 (37.7) | 2,494 (37.7) |
| Diploma or higher | 554 (6.5) | 461 (7.0) | 461 (7.0) |
| Occupation, n (%) |  |  |  |
| Unemployed | 3,571 (41.6) | 3,000 (45.4) | 3,000 (45.4) |
| Student | 92 (1.1) | 73 (1.1) | 73 (1.1) |
| Employed | 4,929 (57.4) | 3,535 (53.5) | 3,535 (53.5) |
| Rural area, n (%) | 4,100 (47.7) | 2,886 (43.7) | 2,886 (43.7) |
|  |  |  |  |
| ***Clinical parameters*** |  |  |  |
| Blood pressure (mg/dL), mean (SD) |  |  |  |
| Systolic | 143.6 (25.6) | 143.8 (26.0) | 143.8 (26.0) |
| Diastolic | 90.7 (13.3) | 91.5 (13.6) | 91.5 (13.6) |
| Triglycerides (mg/dL), mean (SD) | 174.1 (115.5) | 165.7 (111.9) | 165.7 (111.9) |
| High-density lipoprotein (mg/dL), mean (SD) | 43.2 (9.6) | 44.3 (9.7) | 44.3 (9.7) |
| Low-density lipoprotein (mg/dL), mean (SD) | 134.3 (36.3) | 136.3 (36.4) | 136.3 (36.4) |
| Total cholesterol (mg/dL), mean (SD) | 197.0 (43.1) | 198.8 (43.0) | 198.8 (43.0) |
| Fasting plasma glucose (mg/dL), mean (SD) | 117.0 (46.5) | 116.0 (45.6) | 116.0 (45.6) |
|  |  |  |  |
| ***Modified FINDRISC components*** |  |  |  |
| Age (years), mean (SD) | 48.4 (13.0) | 47.7 (12.5) | 47.7 (12.5) |
| Body mass index (kg/m2), mean (SD) | 27.1 (4.8) | 28.4 (4.3) | 28.4 (4.3) |
| Waist circumference (cm), mean (SD) | 88.8 (11.6) | 92.7 (9.5) | 92.7 (9.5) |
| Daily physical activity, no, n (%) | 1,680 (19.6) | 1,141 (17.3) | 1,141 (17.3) |
| Fruits and vegetables consumption, no, n (%) | 3,086 (35.9) | 2,286 (34.6) | 2,286 (34.6) |
| Antihypertensive medication, yes, n (%) | 3,566 (41.5) | 2,881 (43.6) | 2,881 (43.6) |
| History of hyperglycaemia, yes, n (%) | 847 (9.9) | 661 (10.0) | 661 (10.0) |
| Family with diabetes, yes, n (%) | 361 (4.2) | 303 (4.6) | 303 (4.6) |
| Modified FINDRISC score, mean (SD) | 8.6 (3.9) | 9.9 (3.2) | 9.9 (3.2) |

*Notes.* NCEP-ATP III, National Cholesterol Education Program Adult Treatment Panel III; IDF, International Diabetes Federation; SD, standard deviation.
