# Supplementary material for: Accuracy of the Modified Finnish Diabetes Risk Score (Modified FINDRISC) for detecting metabolic syndrome: Findings from the Indonesian national health survey
Source: PLoS One. 2025 Feb 12;20(2):e0314824. doi: 10.1371/journal.pone.0314824 (PMC11819590; doi:10.1371/journal.pone.0314824)
Supplement: S6 Table — (DOCX) [file pone.0314824.s007.docx]

**S6 Table.** Different cut-offs and diagnostic accuracy of Modified FINDRISC to detect individuals with metabolic syndrome in Indonesia

| Cut-off | NCEP-ATP III | | | | | | | |  | Cut-off | IDF | | | | | | | |
| --- | --- | --- | --- | --- | --- | --- | --- | --- | --- | --- | --- | --- | --- | --- | --- | --- | --- | --- |
|  | Sen | Spe | PPV | NPV | Youden index | Case above the cut-off (%) | Case detected (%) | NNS |  |  | Sen | Spe | PPV | NPV | Youden index | Case above the cut-off  (%) | Case detected (%) | NNS |
| 1 | 96.4 | 26.3 | 40.0 | 93.5 | 0.23 | 81.4 | 96.4 | 2.5 |  | 1 | 100.0 | 25.2 | 31.9 | 100.0 | 0.25 | 81.4 | 100.0 | 3.1 |
| 2 | 94.2 | 37.5 | 43.5 | 92.7 | 0.32 | 73.2 | 94.2 | 2.3 |  | 2 | 100.0 | 36.2 | 35.5 | 100.0 | 0.36 | 73.2 | 100.0 | 2.8 |
| 3 | 90.8 | 49.2 | 47.7 | 91.3 | 0.40 | 64.3 | 90.8 | 2.1 |  | 3 | 100.0 | 48.3 | 40.4 | 100.0 | 0.48 | 64.3 | 100.0 | 2.5 |
| 4 | 85.9 | 58.9 | 51.6 | 89.1 | 0.45 | 56.2 | 85.9 | 1.9 |  | 4 | 97.9 | 58.4 | 45.2 | 98.7 | 0.56 | 56.2 | 97.9 | 2.2 |
| 5 | 79.6 | 68.7 | 56.5 | 86.9 | 0.48 | 47.6 | 79.6 | 1.8 |  | 5 | 93.9 | 68.6 | 51.2 | 97.0 | 0.63 | 47.6 | 93.9 | 2.0 |
| **6** | **74.0** | **75.5** | **60.6** | **85.1** | **0.50** | **41.2** | **74.0** | **1.6** |  | **6** | **89.8** | **75.8** | **56.6** | **95.5** | **0.66** | **41.2** | **89.8** | **1.8** |
| 7 | 60.4 | 83.7 | 65.3 | 80.5 | 0.44 | 31.2 | 60.4 | 1.5 |  | 7 | 74.2 | 83.8 | 61.7 | 90.2 | 0.58 | 31.2 | 74.2 | 1.6 |
| 8 | 51.1 | 88.3 | 69.1 | 78.0 | 0.39 | 25.0 | 51.1 | 1.4 |  | 8 | 63.4 | 88.5 | 65.9 | 87.3 | 0.52 | 25.0 | 63.4 | 1.5 |
| 9 | 38.9 | 92.3 | 72.1 | 74.8 | 0.31 | 18.2 | 38.9 | 1.4 |  | 9 | 48.6 | 92.4 | 69.3 | 83.7 | 0.41 | 18.2 | 48.6 | 1.4 |
| 10 | 29.5 | 94.9 | 74.8 | 72.5 | 0.24 | 13.3 | 29.5 | 1.3 |  | 10 | 37.0 | 95.0 | 72.1 | 81.1 | 0.32 | 13.3 | 37.0 | 1.4 |
| 11 | 20.9 | 96.8 | 77.1 | 70.6 | 0.18 | 9.2 | 20.9 | 1.3 |  | 11 | 26.4 | 96.9 | 74.8 | 78.9 | 0.23 | 9.2 | 26.4 | 1.3 |
| 12 | 14.1 | 98.3 | 80.6 | 69.1 | 0.12 | 5.9 | 14.1 | 1.2 |  | 12 | 17.8 | 98.3 | 78.7 | 77.3 | 0.16 | 5.9 | 17.8 | 1.3 |
| 13 | 10.1 | 99.0 | 84.1 | 68.3 | 0.09 | 4.1 | 10.1 | 1.2 |  | 13 | 12.9 | 99.0 | 82.5 | 76.4 | 0.12 | 4.1 | 12.9 | 1.2 |
| 14 | 6.5 | 99.4 | 85.1 | 67.6 | 0.06 | 2.6 | 6.5 | 1.2 |  | 14 | 8.4 | 99.4 | 83.8 | 75.6 | 0.08 | 2.6 | 8.4 | 1.2 |
| 15 | 4.7 | 99.7 | 88.3 | 67.2 | 0.04 | 1.8 | 4.7 | 1.1 |  | 15 | 6.1 | 99.7 | 87.4 | 75.1 | 0.06 | 1.8 | 6.1 | 1.1 |
| 16 | 3.1 | 99.8 | 89.2 | 66.9 | 0.03 | 1.2 | 3.1 | 1.1 |  | 16 | 3.9 | 99.8 | 88.1 | 74.7 | 0.04 | 1.2 | 3.9 | 1.1 |
| 17 | 1.7 | 99.9 | 90.4 | 66.6 | 0.02 | 0.7 | 1.7 | 1.1 |  | 17 | 2.2 | 99.9 | 89.2 | 74.4 | 0.02 | 0.7 | 2.2 | 1.1 |
| 18 | 0.9 | 100.0 | 91.6 | 66.4 | 0.01 | 0.3 | 0.9 | 1.1 |  | 18 | 1.2 | 100.0 | 91.6 | 74.2 | 0.01 | 0.3 | 1.2 | 1.1 |
| 19 | 0.4 | 100.0 | 92.5 | 66.3 | 0.00 | 0.2 | 0.4 | 1.1 |  | 19 | 0.6 | 100.0 | 92.5 | 74.1 | 0.01 | 0.2 | 0.6 | 1.1 |
| 20 | 0.2 | 100.0 | 94.4 | 66.3 | 0.00 | 0.1 | 0.2 | 1.1 |  | 20 | 0.3 | 100.0 | 94.4 | 74.1 | 0.00 | 0.1 | 0.3 | 1.1 |
| 21 | 0.1 | 100.0 | 90.9 | 66.2 | 0.00 | 0.0 | 0.1 | 1.1 |  | 21 | 0.2 | 100.0 | 90.9 | 74.0 | 0.00 | 0.0 | 0.2 | 1.1 |
| 22 | 0.1 | 100.0 | 100.0 | 66.2 | 0.00 | 0.0 | 0.1 | 1.0 |  | 22 | 0.1 | 100.0 | 100.0 | 74.0 | 0.00 | 0.0 | 0.1 | 1.0 |
| 23 | 0.0 | 100.0 | 100.0 | 66.2 | 0.00 | 0.0 | 0.0 | 1.0 |  | 23 | 0.0 | 100.0 | 100.0 | 74.0 | 0.00 | 0.0 | 0.0 | 1.0 |
| 24 | 0.0 | 100.0 | 100.0 | 66.2 | 0.00 | 0.0 | 0.0 | 1.0 |  | 24 | 0.0 | 100.0 | 100.0 | 74.0 | 0.00 | 0.0 | 0.0 | 1.0 |

*Notes***.** NCEP-ATP III: National Cholesterol Education Program Adult Treatment Panel III; IDF: International Diabetes Federation; PPV: positive predictive value; NPV: negative predictive value; NNS: number needed to screen; Sen: sensitivity, Spe: specificity.
