# Supplementary material for: Accuracy of the Modified Finnish Diabetes Risk Score (Modified FINDRISC) for detecting metabolic syndrome: Findings from the Indonesian national health survey
Source: PLoS One. 2025 Feb 12;20(2):e0314824. doi: 10.1371/journal.pone.0314824 (PMC11819590; doi:10.1371/journal.pone.0314824)
Supplement: S7 Table — (DOCX) [file pone.0314824.s008.docx]

**S7 Table.** The comparison of diagnostic accuracy of Modified FINDRISC to detect metabolic syndrome in different age groups

|  | AUC, % | | | |
| --- | --- | --- | --- | --- |
|  | Age < 35 | Age 35-64 | Age >64 | Total participants |
| NCEP-ATP III | 82.1 (80.7-83.5) | 79.8 (79.1-80.4) | 77.6 (75.6-79.5) | 80.9 (80.3-81.5) |
| IDF | 91.4 (90.7-92.1) | 88.2 (87.7-88.7) | 92.7 (91.8-93.6) | 88.9 (88.5-89.3) |

*Notes*. NCEP-ATP III: National Cholesterol Education Program Adult Treatment Panel III; IDF: International Diabetes Federation: AUC: area under the receiver operating characteristic curve.
