# Supplementary material for: Accuracy of the Modified Finnish Diabetes Risk Score (Modified FINDRISC) for detecting metabolic syndrome: Findings from the Indonesian national health survey
Source: PLoS One. 2025 Feb 12;20(2):e0314824. doi: 10.1371/journal.pone.0314824 (PMC11819590; doi:10.1371/journal.pone.0314824)
Supplement: S1 File — (DOCX) [file pone.0314824.s009.docx]

**S1 File.** Research protocol.

**Accuracy of the Finnish Diabetes Risk Score (FINDRISC) Instrument to Detect Individuals with Dysglycaemia, Undiagnosed Type 2 Diabetes, and**

**Metabolic Syndrome**

**SUMMARY**

The Finnish Diabetes Risk Score (FINDRISC) was initially developed as an instrument to predict individuals' risk of developing diabetes in the future. Lately, FINDRISC is also used as an instrument to identify individuals with prediabetes, dysglycaemia, type 2 diabetes, and metabolic syndrome.

A previous study has translated, adapted, and validated FINDRISC for the Indonesian population (FINDRISC-Indonesian), particularly in Java and Sulawesi. The use of FINDRISC for screening can help optimize the screening budget for non-communicable diseases, especially type 2 diabetes. The screening process can be conducted using a two-stage approach, starting with FINDRISC-Indonesian as the initial screening tool, followed by blood glucose checks for individuals identified as high-risk groups based on FINDRISC scores.

Additional evidence and external validation are required by evaluating the diagnostic accuracy of FINDRISC as a screening tool on other datasets outside the dataset that has been used for the initial adaptation of FINDRISC to the Indonesian population, such as the Riskesdas 2018 dataset. Therefore, the diagnostic accuracy of FINDRISC-Indonesian for detecting individuals with dysglycaemia, type 2 diabetes, and metabolic syndrome in the Riskesdas 2018 dataset will provide further crucial evidence to support recommendations for the use of this instrument in the Indonesian population.

1. **BACKGROUND**

The International Diabetes Federation (IDF) ranked Indonesia as the 6^th^ country with the highest number of diabetes patients, with approximately 10.3 million diabetes patients in 2017^1^. The Indonesian Ministry of Health reported that new cases of type 2 diabetes have doubled from 1.1% in 2007 to 2.1% in 2013^2^. Therefore, diabetes is considered a high burden disease for both patients, families, and the healthcare system^3^. In addition, type 2 diabetes is the most common type of diabetes, and 90% of diabetes cases are type 2 diabetes^1,2^.

A report from WHO shows that the number of diabetes sufferers globally will continue to increase if no effective prevention programs are introduced. Scientific evidence shows that preventing or delaying the progression of type 2 diabetes can be done through lifestyle modifications or pharmacological interventions^4,5^. However, type 2 diabetes often remains undetected for years until complications appear^6^. Effective interventions for the prevention of type 2 diabetes are also difficult to implement in all populations^7^. Therefore, it is very important to design an effective program to screen groups at high risk of developing type 2 diabetes and put them in diabetes prevention programs^8,9^.

Early detection or screening of individuals at high risk of developing type 2 diabetes through cost-effective, non-invasive and reliable procedures is very crucial. This is mainly to increase their awareness and change their behavior and lifestyle to prevent further risk of diabetes^7^. The currently recommended screening method is by using blood glucose levels, such as fasting blood glucose (FBG) or oral glucose tolerance test (OGTT). However, both screening methods have challenges, especially in terms of logistics and expensive costs compared to screening methods based on risk scores^10^.

The Finnish Diabetes Risk Score (FINDRISC) can be utilized to identify individuals at high risk of developing type 2 diabetes. It has been proven to be reliable, valid, practical, and has been studied in various countries^10–22^. In addition, the FINDRISC can be used to predict individuals with undiagnosed dysglycemia and type 2 diabetes^22^. The FINDRISC-Indonesian is the translated version of FINDRISC which has undergone through a cross-sectional adaptation and validation process in the Indonesian population^23^. The FINDRISC-Indonesian has a receiver-operating characteristic (ROC) area of 0.7-0.8. Several previous studies have shown that FINDRISC can also be used to detect individuals with metabolic syndrome^11,24^, but there have been no similar studies in Indonesia.

The Indonesian Ministry of Health conducts basic health research (Riskesdas) every 5 years, and the latest data available is from Riskesdas 2018^25^. Before the FINDRISC-Indonesian can be recommended in Indonesia, it needs to undergo external validation by evaluating the diagnostic accuracy of FINDRISC as a screening instrument on other datasets, such as Riskesdas 2018 dataset. This information can provide important additional evidence to support recommendations for the use of this instrument in the Indonesian population. This external validation is necessary to provide further evidence of the effectiveness of a two-stage screening approach, where FINDRISC-Indonesian as the initial screening approach followed by blood glucose checks, in order to increase coverage and the cost-benefit ratio of the screening program.

1. **OBJECTIVES**

The research objectives are as follows:

1. To determine the diagnostic accuracy of FINDRISC-Indonesian diagnostics in identifying individuals with dysglycemia and type 2 diabetes who have not been previously diagnosed with diabetes in the Riskesdas 2018 dataset.

2. To determine the diagnostic accuracy of the FINDRISC-Indonesian diagnosis in identifying individuals at risk of metabolic syndrome in the Riskesdas 2018 dataset.

1. **METHODS**

The dataset that will be used in this study is the 2018 Riskesdas dataset. For the first research objective, the inclusion criteria are participants who have blood glucose data. Exclusion criteria are participants who have been diagnosed with diabetes mellitus (either type 1 or 2) or are using medication that can lower blood glucose levels. For the second research objective, the inclusion criteria are participants who have data on triglycerides, HDL, blood pressure and blood glucose levels.

**FINDRISC-Indonesian**

FINDRISC consists of 8 questions, namely age, BMI (kg/m^2^), waist circumference (cm), physical activity, vegetable and fruit consumption, use of anti-hypertensive drugs, history of high blood glucose, and family history of diabetes. The FINDRISC instrument has been translated and validated on 1,403 participants from Java and Sulawesi to detect participants with undetected dysglycemia and type 2 diabetes^23^.

The scoring from the FINDRISC-Indonesian will follow the validation results that have been carried out in Indonesia^23^. Apart from that, the scoring can be adjusted to the variables in the 2018 Riskesdas, as has been done in other research by adjusting the FINDRISC scoring according to the existing variables^26^.

**Definition of dysglycemia, undiagnosed type 2 diabetes, and risk of metabolic syndrome**

Dysglycemia and undiagnosed type 2 diabetes will be defined based on guidelines from the Indonesian Endocrinology Association (Perkeni)^27^. For example, if based on fasting blood glucose (FBG) levels, participants can be grouped as normal (FBG <100mg/dL), prediabetes (100-125mg/dL) and type 2 diabetes (>126mg/dL). Dysglycemia is a combination of participants with prediabetes and type 2 diabetes.

Metabolic syndrome can be determined based on the National Cholesterol Education Program Adult Treatment Panel III (NCEP-ATP III) and IDF criteria. Based on NCEP-ATP III, metabolic syndrome can be defined if the patient has at least 3 of the following characteristics: (1) central obesity, (2) triglycerides >150mg/dl, (3) HDL <40mg/dl for men or <50mg/dl for women, (4) blood pressure >130/85mmHg or using antihypertensive drugs, and (5) fasting blood glucose >100mg/dl. Based on IDF criteria, participants are considered to have metabolic syndrome if they have central obesity and meet at least two of the following criteria: (1) triglycerides ≥150 mg/dl, (2) HDL <40 mg/dl for men and <50 mg/dl for women, (3) systolic blood pressure ≥130 or diastolic blood pressure ≥85 mmHg or use antihypertensive medications, and (4) fasting blood glucose ≥100 mg/dl or using glucose-lowering medications.

**Required Data**

The data that is required includes sociodemographic data, FINDRISC-Indonesian score, blood glucose level data (HbA1C, fasting blood glucose, random blood glucose, or OGTT). For the second objective, additional data needed are triglycerides, HDL, and blood pressure. A summary of the required data in Riskesdas 2018 for each research objective can be seen in Table 1.

1. **DATA ANALYSIS**

Categorical data will be described as n (%), while continuous data will be displayed as mean (SD). The different tests will be adjusted according to the type of data, normality tests, and the number of groups being compared. To test the differences in categorical data, the Chi-square statistical analysis or Fisher's test will be used. For continuous data that follows a normal distribution, the independent t-test will be used if there are two groups, or the ANOVA test for more than two groups. For continuous data that does not follow a normal distribution, the Mann-Whitney test will be used for two groups, or Kruskal-Wallis for more than two groups. The normality test will be conducted using the QQ plot test and the Kolmogorov-Smirnov test.

The diagnostic accuracy will be analyzed using the receiver-operating characteristic (ROC) curve. Additionally, sensitivity, specificity, false positives, false negatives, positive predictive value, negative predictive value, and the percentage of participants requiring confirmation of the measurement through additional blood glucose examination will be analyzed.

Data analysis will be carried out using SPSS version 26.0 or R software. The significance level (*p*-value) used will be 0.05.

Table 1. Required data in Riskesdas 2018

| No | Objective | Required data |
| --- | --- | --- |
| 1 | Research objective 1 | - Sociodemography |
|  |  | - age |
|  |  | - gender |
|  |  | - education level |
|  |  | - occupation |
|  |  | - province |
|  |  | - residential area (urban/rural) |
|  |  | - FINDRISC Score-Indonesian |
|  |  | - Age (years) |
|  |  | - BMI (kg/m^2^) |
|  |  | - waist circumference (cm) |
|  |  | - physical activity |
|  |  | - vegetables and fruit consumption |
|  |  | - use of anti-hypertensive drugs |
|  |  | - history of high blood glucose |
|  |  | - family history of diabetes |
|  |  | - Smoking habit |
|  |  | - Upper arm circumference |
|  |  | - History of diabetes |
|  |  | - Blood glucose level |
| 2 | Research objective 2 | - Sociodemography |
|  |  | - FINDRISC Score-Indonesian |
|  |  | - Patient clinical data to estimate metabolic syndrome status |
|  |  | - blood glucose level |
|  |  | - blood pressure |
|  |  | - HDL, LDL, total cholesterol levels |
|  |  | - triglycerides |

*Notes.* FINDRSC Indonesian has been validated by previous research^23^

**REFERENCES**

1. IDF. *IDF Diabetes Atlas Eighth Edition 2017*. 8th ed.; 2017. doi:http://dx.doi. org/10.1016/S0140-6736(16)31679-8.

2. Pusdatin. *Situasi Dan Analisis Diabetes*. Kementerian Kesehatan Republik Indonesia; 2014. doi:10.1002/ajmg.a.35913

3. Valensi P, Schwarz EH, Hall M, Felton AM, Maldonato A, Mathieu C. Pre-diabetes essential action: A European perspective. *Diabetes Metab*. 2005;31(6):606-620. doi:10.1016/S1262-3636(07)70239-2

4. Knowler WC, Barrett-Connor E, Fowler SE, et al. Reduction in the incidence of type 2 diabetes with lifestyle intervention or metformin. *N Engl J Med*. 2002;346(6):393-403. doi:10.1056/NEJMoa012512

5. Pan X-R, Li G-W, Hu Y-H, et al. Effects of Diet and Exercise in Preventing NIDDm in People with Impaired GLucose Tolerance. *Diabetes Care*. 1997;22(4):537-544. doi:10.1007/BF01899717

6. World Health Organization. *Global Report on Diabetes.* WHO; 2016. doi:10.1128/AAC.03728-14

7. Milovanovic S, Silenzi A, Kheiraoui F, Ventriglia G, Boccia S, Poscia A. Detecting persons at risk for diabetes mellitus type 2 using FINDRISC: results from a community pharmacy-based study. *Eur J Public Health*. 2018;28(6):1127-1132. doi:10.1093/eurpub/cky009

8. Schwarz PEH, Schwarz J, Bornstein SR, Schulze J. Diabetes prevention - From physiology to implementation. *Horm Metab Res*. 2006;38(7):460-464. doi:10.1055/s-2006-947839

9. Schwarz PEH, Schwarz J, Schuppenies A, Bornstein SR, Schulze J. Development of A Diabetes Prevention Management Program for Clinical Practice. *Public Health Rep*. 2007;122:258-263. doi:10.1177/003335490612100617

10. Schulze J, Bornstein S, Schwarz P, Bergmann A, Wang L, Li J. A Simplified Finnish Diabetes Risk Score to Predict Type 2 Diabetes Risk and Disease Evolution in a German Population. *Horm Metab Res*. 2007;39(9):677-682. doi:10.1055/s-2007-985353

11. Makrilakis K, Liatis S, Grammatikou S, et al. Validation of the Finnish diabetes risk score (FINDRISC) questionnaire for screening for undiagnosed type 2 diabetes, dysglycaemia and the metabolic syndrome in Greece. *Diabetes Metab*. 2010;37(2):144-151. doi:10.1016/j.diabet.2010.09.006

12. Bernabe-Ortiz A, Perel P, Miranda JJ, Smeeth L. Diagnostic accuracy of the Finnish Diabetes Risk Score (FINDRISC) for undiagnosed T2DM in Peruvian population. *Prim Care Diabetes*. 2018;12(6):517-525. doi:10.1016/j.pcd.2018.07.015

13. Štiglic G, Fijačko N, Stožer A, Sheikh A, Pajnkihar M. Validation of the Finnish Diabetes Risk Score (FINDRISC) questionnaire for undiagnosed type 2 diabetes screening in the Slovenian working population. *Diabetes Res Clin Pract*. 2016;120:194-197. doi:10.1016/j.diabres.2016.08.010

14. Lee W, Chisholm H, Jiang Y, Poppitt SD, Volkova K, Silvestre MP. Evaluating FINDRISC as a screening tool for type 2 diabetes among overweight adults in the PREVIEW:NZ cohort. *Prim Care Diabetes*. 2017;11(6):561-569. doi:10.1016/j.pcd.2017.07.003

15. Soriguer F, Valdés S, Tapia MJ, et al. Validación del FINDRISC (FINnish Diabetes Risk SCore) para la predicción del riesgo de diabetes tipo 2 en una población del sur de España. Estudio Pizarra. *Med Clin (Barc)*. 2012;138(9):371-376. doi:10.1016/j.medcli.2011.05.025

16. A Ishaque, corresponding author1 F Shahzad, 2 FH Muhammad, 3 Y Usman 4 and Z Ishaque5. Diabetes risk assessment among squatter settlements in Pakistan: A cross-sectional study. *Malays Fam Physician*. 2016;11(2):2-3.

17. Barengo NC, Tamayo DC, Tono T, Tuomilehto J. A Colombian diabetes risk score for detecting undiagnosed diabetes and impaired glucose regulation. *Prim Care Diabetes*. 2017;11(1):86-93. doi:10.1016/j.pcd.2016.09.004

18. Salinero-Fort MA, Burgos-Lunar C, Lahoz C, et al. Performance of the Finnish Diabetes Risk Score and a simplified Finnish Diabetes Risk Score in a community-based, cross-sectional programme for screening of undiagnosed type 2 diabetes mellitus and dysglycaemia in Madrid, Spain: The SPREDIA-2 study. *PLoS One*. 2016;11(7):1-17. doi:10.1371/journal.pone.0158489

19. Zhang M, Zhang H, Wang C, et al. Development and validation of a risk-score model for type 2 diabetes: A cohort study of a rural adult Chinese population. *PLoS One*. 2016;11(4):1-13. doi:10.1371/journal.pone.0152054

20. Guasch-Ferré M, Bulló M, Costa B, et al. A risk score to predict type 2 diabetes mellitus in an elderly spanish mediterranean population at high cardiovascular risk. *PLoS One*. 2012;7(3):2-8. doi:10.1371/journal.pone.0033437

21. Dugee O, Janchiv O, Jousilahti P, et al. Adapting existing diabetes risk scores for an Asian population: A risk score for detecting undiagnosed diabetes in the Mongolian population. *BMC Public Health*. 2015;15(1):1-9. doi:10.1186/s12889-015-2298-9

22. Mavrogianni C, Lambrinou CP, Androutsos O, et al. Evaluation of the Finnish Diabetes Risk Score as a screening tool for undiagnosed type 2 diabetes and dysglycaemia among early middle-aged adults in a large-scale European cohort. The Feel4Diabetes-study. *Diabetes Res Clin Pract*. 2019;150:99-110. doi:10.1016/j.diabres.2019.02.017

23. Rokhman MR, Arifin B, Zulkarnain Z, et al. Translation and validation of the Finnish Diabetes Risk Score for detecting undiagnosed diabetes and dysglycaemia in the Indonesian population. *Manuscr Submitt Publ*. Published online 2021.

24. Janghorbani M, Adineh H, Amini M. Evaluation of the Finnish Diabetes Risk Score (FINDRISC) as a screening tool for the metabolic syndrome. *Rev Diabet Stud*. 2013;10(4):283-292. doi:10.1900/RDS.2013.10.283

25. Indonesia Ministry of Health. *National Basic Health Survey*. Indonesia Ministry of Health; 2018. doi:1 Desember 2013

26. Kulkarni M, Foraker RE, McNeill AM, et al. Evaluation of the Modified FINDRISC Diabetes Score to Identify Individuals at High Risk for Diabetes among Middle-aged White and Black ARIC Study Participants. *Diabetes Obes Metab*. 2017;19(9):1260-1266. doi:10.1111/dom.12949

27. Tim Penyusun. *Pedoman Pengelolaan Dan Pencegahan Diabetes Melitus Tipe 2 Dewasa Di Indonesia*. PB Perkeni; 2019.
